# Element-Doped Functional Carbon-Based Materials

**DOI:** 10.3390/ma13020333

**Published:** 2020-01-11

**Authors:** Sergio Morales-Torres, Agustín F. Pérez-Cadenas, Francisco Carrasco-Marín

**Affiliations:** Carbon Materials Research Group, Department of Inorganic Chemistry, Faculty of Sciences, University of Granada, Avenida de Fuente Nueva, s/n, ES18071 Granada, Spain; afperez@ugr.es (A.F.P.-C.); fmarin@ugr.es (F.C.-M.)

**Keywords:** carbon materials, heteroatoms, doping, surface chemistry, adsorption, catalysis, environmental remediation, energy storage

## Abstract

Carbon materials are one of the most fascinating materials because of their unique properties and potential use in several applications. They can be obtained from agricultural waste, organic polymers, or by using advanced synthesizing technologies. The carbon family is very wide, it includes classical activated carbons to more advanced types like carbon gels, graphene, and so on. The surface chemistry of these materials is one of the most interesting aspects to be studied. The incorporation of different types of chemical functionalities and/or heteroatoms such as O, N, B, S, or P on the carbon surface enables the modification of the acidic–basic character, hydrophilicity–hydrophobicity, and the electron properties of these materials, which in turn determines the final application. This book collects original research articles focused on the synthesis, properties, and applications of heteroatom-doped functional carbon materials.

The broad family of carbon materials includes classical activated carbons to carbon nanostructures like carbon gels, carbon nanotubes, fullerenes, graphene, and so on. In general, these materials present different properties and origins, but all of them possess a common characteristic, in other words, the ability to be prepared in many different shapes such as pellets, granular, powders, cloths, fibers, monoliths, foams, coatings, films, and so on. Furthermore, their porous texture and chemical properties can be tailored by physical/thermal and chemical processes, enabling the development of porosity and specific surface area and the incorporation of different chemical functionalities. Both porosity and surface chemistry have a marked influence on their performance in a specific application, either by themselves or in combination with other materials. In fact, carbon materials have demonstrated to be excellent options as adsorbents [1], catalysts [2,3], or catalyst supports [4] when compared to classic materials (e.g., alumina, silica or ceria) as consequence of their high stability in both acidic and alkaline media.

Surface chemistry is the most attractive property of carbon materials, since the chemical groups anchored on the carbon surface may interact with organic molecules, inorganic salts, and metals. The most common heteroatoms are oxygen (O), nitrogen (N), sulfur (S), boron (B), and phosphorus (P). They are often part of functional groups and determine the acidic–basic character and the hydrophilicity–hydrophobicity [5,6,7]. For instance, oxygen-containing groups such as carboxylic acids, anhydrides, lactones, and phenols have an acidic character, while quinones, pyrones, and chromene are basic groups [8,9,10]. On the other hand, delocalized π electrons from the basal planes also contribute to the basicity [11], but also to the variation of the electron density. This effect can also be achieved by the incorporation of boron atoms or nitrogen-containing groups (i.e., pyridine and pyrrole), and deficient or additional electrons being provided, respectively. Thus, changes in the chemical properties of carbon materials influence their adsorption behavior and catalytic activity in some reactions [1,4].

This Special Issue deals with the recent advances in heteroatom-doped carbon materials. Different synthesis procedures, characterization techniques, and applications were investigated for these functional materials. The Special Issue collects eleven full-length articles and a short communication.

H. Hamad et al. [12] prepared carbon–phosphorus–titanium composites from cellulose to be used as photocatalysts in the removal of Orange-G dye. They pointed out that the phosphorus-containing groups incorporated in the composites modified their textural properties, crystallinity, and photocatalytic performance. S. Zhang et al. [13] modified biochars obtained from agricultural waste using 3-mercaptopropyltrimethoxysilane epoxy-chloropropane via an ionic-imprinted technique. These materials were active as adsorbents of Cd (II) in an aqueous solution, showing a higher Cd-selectivity in the presence of Co (II), Pb (II), Zn (II), and Cu (II) and a good stability after several adsorption–desorption cycles. A. Elmouwahidi et al. [14] developed carbon materials from waste woods by KOH activation. The surface chemistry was modified by different chemical agents, which incorporated nitrogen- and oxygen-containing groups on the carbon surface. All doped materials, with the exception of that treated with nitric acid, showed good capacitance values and high cyclic stability when used as electrodes for supercapacitors. An alternative method to obtain N-doped carbon materials for the same application was proposed by T. Ai. et al. [15]. This method consisted of the use of a N-containing bio-phenolic resin as a precursor and subsequent activation by a molten-salt method. Carbon materials have also been demonstrated to be efficient electrocatalysts in the oxygen reduction reaction (ORR). A. Abdelwahab et al. [16] studied Co- and Ni-doped carbon xerogels, while N-doped carbon fibers and microspheres synthesized from apricot sap were proposed by R. Kanuragaran et al. [17].

Carbon capture is a growing technology, whose implementation can be achieved by the research of novel materials. R. Wei et al. [18] prepared N-doped carbon materials from resorcinol and formaldehyde after KOH activation and ammonia carbonization. A. A. Alghamdi et al. [19] employed N-doped graphene oxide sheets (N-GOs) obtained from different N-containing polymers and after KOH activation. In general, the CO_2_ capture capacity by N-doped materials was enhanced by the increase of the nitrogen content, the surface area, and the micropore volume. E. Rodriguez-Acevedo et al. [20] demonstrated that shallow reservoirs could be effective for carbon capture after injecting nanofluids based on N-rich carbon nanospheres. Finally, the last articles of this Special Issue deal with the development of N-doped graphene films for high sensitivity electrodes [21]; the functionalization of graphene oxides with p-phenylenediamine as a modifier [22]; and the induction of magnetic moments in graphene by introducing sp^3^-defects [23].

All the published papers were strictly peer reviewed following the standard review practices for the Materials journal. As the Guest Editors of this Special Issue, we acknowledge all of the authors for their prime contributions and the reviewers for their valuable comments to improve the quality of the papers. Finally, we would like to thank the staff members of Materials, in particular Clark Xu for its kind assistance.
